# Special Issue “Molecular Simulation and Modeling”

**DOI:** 10.3390/ijms26051924

**Published:** 2025-02-23

**Authors:** Małgorzata Borówko

**Affiliations:** Department of Theoretical Chemistry, Institute of Chemical Sciences, Faculty of Chemistry, Maria Curie-Skłodowska University in Lublin, 20-031 Lublin, Poland; malgorzata.borowko@mail.umcs.pl

Molecular simulation and modeling have a huge and growing impact on scientific progress. We are witnessing a kind of “transfer of experience to cyberspace moving”. This became possible thanks to the development of advanced theories, new computational methods, and, above all, thanks to the unimaginable increase in the power of modern computers [1,2,3,4].

Let us imagine a triangle whose vertices contain basic research methods: experiment, theory, and simulation. Such a triangle is a good representation of modern research, which requires a combination of different methods. There is still a widespread view in the natural sciences that experimental confirmation of a scientific hypothesis is crucial. However, an experiment may be difficult or impossible to perform, dangerous, or simply too expensive. Currently, research often focuses on the mechanisms of various phenomena and processes. It is generally difficult to elucidate these mechanisms using experimental techniques alone. In such a situation, it becomes necessary to use “molecular simulation and modeling”. These methods can be supported by the application of machine learning [5,6], expert systems [7], and artificial intelligence [8].

Theoretical research includes both quantum mechanics and classical physics. “Hybrid models” are usually used in computer simulations. M. Karplus, M. Levitt, and A. Warshel developed multiscale models for complex chemical systems in which the central part of the system was described using quantum models, while the surrounding was treated in the framework of classical physics [9]. Typically, “pencil” theories used include quantum computing, statistical thermodynamics, and various phenomenological methods. Although these methods still lead to many significant achievements, molecular simulations are becoming the dominant tool in theoretical research. The fact that scientists can now use computers to conduct “experiments” has provided much deeper insights into the mechanisms of many natural and technological processes. The strength of simulations is that they are universal and can be used for the study of various phenomena.

Today, molecular simulations are standard methods used by researchers in various disciplines, including physics, chemistry, biology, medicine, and engineering. The current state of the art in molecular simulations is discussed in numerous reviews [1,2,3,4]. Molecular simulations are usually performed using the Monte Carlo method or molecular dynamics. The latter is likely the most popular simulation method. This is due to the availability of numerous ready-made software packages that provide core molecular dynamics codes together with rich collections of programs to facilitate the writing of simulation scripts. The molecular dynamics method is now becoming easier and more accessible to users who do not need to have a deeper knowledge of computer science.

Applications of molecular simulations include a rational design of new chemical compounds, macromolecules, nanoparticles, and various assembled structures at any level of organization, from molecules to viruses. They are increasingly used to model new nanomaterials and catalysts, as well as for the optimization of energy storage and solar cells, among others [10,11,12,13]. Although the simulations are used in many fields, the most spectacular results have been achieved in biochemistry and biology [10,11,14]. One can say that the simulations changed the paradigm of structural bioinformatics from the study of single structures to the analysis of conformational ensembles. They reflect the behavior of biomolecules in full atomic detail and at very fine temporal resolutions. Moreover, the methods have appeared to be very valuable in deciphering the functional mechanisms of biomolecules, exploring the structural basis for disease, as well as in the design of desired chemical compounds. Molecular simulation and modeling are also used for molecular docking, allosteric regulation, structure refinement, drug discovery, and many others.

We are now witnessing another civilization leap, which is the possibility of using artificial intelligence in various areas of life and the economy. It also marks a breakthrough in scientific research. For many years, chemists have dreamed of fully controlling the behavior of life proteins’ molecules. We are getting closer to this goal. The Royal Swedish Academy of Sciences decided to award the 2024 Nobel Prize in Chemistry to David Barker “for computational protein design” and jointly to Demis Hassabis and John Jumper for “protein structure prediction” [14]. David Baker has developed computerized methods for creating proteins that did not previously exist and which, in many cases, have entirely new functions. Demis Hassabis and John Jumper have utilized artificial intelligence to predict the structure of almost all known proteins. These methods allow us to better understand how life works, why certain diseases develop or how resistance to antibiotics arises, and to design targeted drugs, faster vaccine development, and minimal sensors—to mention just a few applications.

Groundbreaking discoveries result from the accumulation of knowledge created by many researchers dealing with various, sometimes quite narrow, subjects. The purpose of this Special Issue is to present a wide picture of applications of molecular simulation and modeling, and to draw together contributors from different disciplines to stimulate the exchange of knowledge and experiences. In the collection of articles gathered here, two large groups can be distinguished, devoted to similar issues. The remaining works cover other topics.

The first group of articles is devoted to the modeling of chemical reactions involving very complex molecules. For this purpose, the density functional theory, molecular docking, homology modeling, and molecular dynamics were used. This part of our collection covers various detailed issues: the mechanism of 1,3-dipolar cycloaddition reactions, the affinity of ceftobiprole for selected cyclodextrins, and the prediction of TLR2 heterodimerization.

The papers of the second group deal with various physicochemical processes: adsorption of different nanoparticles on solids, diffusion of alkali in sodium trisilicate, adhesion at the interface of the geopolymer binder and cement mortar, phase transition and thermal deposition of InN, and the opposite spray method for the preparation of CL-20/TNT cocrystal explosives.

The remaining articles cover the following topics: design of new Schiff bases and their heavy metal ion complexes for environmental applications, viral immunogenicity prediction by machine learning methods, a practical guide to all-atom and coarse-grained molecular dynamic simulations using Amber and Gromax (presented on the example of the study of the disulfide-bond impact on the intrinsically discovered amyloid beta), and a review on theoretical models for the prediction of flavonoid oxidation potentials and antioxidant activities.

I hope that all the articles presented here will illustrate the power of molecular simulations and modeling in natural science.

Finally, I would like to thank all authors for their contributions to this Special Issue.
